# Increased renal sodium absorption by inhibition of prostaglandin synthesis during fasting in healthy man. A possible role of the epithelial sodium channels

**DOI:** 10.1186/1471-2369-11-28

**Published:** 2010-10-28

**Authors:** Thomas G Lauridsen, Henrik Vase, Jørn Starklint, Carolina C Graffe, Jesper N Bech, Søren Nielsen, Erling B Pedersen

**Affiliations:** 1 Department of Medical Research, Holstebro Hospital, Lægaardvej 12, 7500 Holstebro, Denmark; 2 University of Aarhus, 8000 Aarhus C, Denmark; 3 The Water and Salt Research Center, Institute of Anatomy, University of Aarhus Aarhus C, Denmark

## Abstract

**Background:**

Treatment with prostaglandin inhibitors can reduce renal function and impair renal water and sodium excretion. We tested the hypotheses that a reduction in prostaglandin synthesis by ibuprofen treatment during fasting decreased renal water and sodium excretion by increased absorption of water and sodium via the aquaporin2 water channels and the epithelial sodium channels.

**Methods:**

The effect of ibuprofen, 600 mg thrice daily, was measured during fasting in a randomized, placebo-controlled, double-blinded crossover study of 17 healthy humans. The subjects received a standardized diet on day 1, fasted at day 2, and received an IV infusion of 3% NaCl on day 3. The effect variables were urinary excretions of aquaporin2 (u-AQP2), the beta-fraction of the epithelial sodium channel (u-ENaCbeta), cyclic-AMP (u-cAMP), prostaglandin E2 (u-PGE2). Free water clearance (CH2O), fractional excretion of sodium (FENa), and plasma concentrations of vasopressin, angiotensin II, aldosterone, atrial-, and brain natriuretic peptide.

**Results:**

Ibuprofen decreased u-AQP2, u-PGE2, and FENa at all parts of the study. During the same time, ibuprofen significantly increased u-ENaCbeta. Ibuprofen did not change the response in p-AVP, u-c-AMP, urinary output, and free water clearance during any of these periods. Atrial-and brain natriuretic peptide were higher.

**Conclusion:**

During inhibition of prostaglandin synthesis, urinary sodium excretion decreased in parallel with an increase in sodium absorption and increase in u-ENaCbeta. U-AQP2 decreased indicating that water transport via AQP2 fell. The vasopressin-c-AMP-axis did not mediate this effect, but it may be a consequence of the changes in the natriuretic peptide system and/or the angiotensin-aldosterone system

**Trial Registration:**

Clinical Trials Identifier: NCT00281762

## Background

Inhibition of prostaglandin synthesis reduces renal water and sodium excretion in the kidneys, especially in patients with renal disease or heart failure. A recent study suggested that increased absorption of sodium took place in the thick ascending limb of Henle during prostaglandin inhibition [1]. However, the distal part of the nephron might also participate in this process, i. e. by an increase in the absorption of water and sodium via the aquaporine2 water channels (AQP2) and epithelial sodium channels (ENaC), respectively. The effect of prostaglandin inhibition on renal water and sodium excretion is most pronounced during conditions with an increased prostaglandin synthesis. During fasting, urinary concentrating and diluting ability was reduced, and AQP2 expression down regulated, possibly due to an antagonizing effect of increased prostaglandin level on the effect of vasopressin on water transport in the principal cells [2-5]. In addition, fasting induced natriuresis in man, and prostaglandin E_2 _inhibited sodium absorption in the collecting ducts in rats and rabbits [6-8]. Thus, elevated renal levels of prostaglandin during fasting might mediate natriuresis via ENaC [9]. The degree of water transport via AQP2 is reflected by the level of urinary excretion of aquaporine2 (u-AQP2) [10]. Correspondingly, the sodium transport via ENaC is supposed to be reflected by level of urinary excretion of the β-fraction of ENaC (u-ENaC_β_). This is the first report with measurement of u-ENaC_β _as a biomarker of the activity of the epithelial sodium channels in the distal tubuli.

In the present study, we measured the effect of inhibition of prostaglandins on u-AQP2 and u-ENaC_β _during fasting. We wanted to test the hypotheses that a reduction of prostaglandin synthesis by ibuprofen treatment during fasting would 1. Increase u-AQP2 and u-ENaC_β _during baseline condition and change the renal response to hypertonic saline infusion, and 2. The increased renal water and sodium absorption during ibuprofen treatment was mediated via increased transport via AQP2 and ENaC.

We performed a randomized, placebo controlled crossover study in healthy humans during baseline condition and during hypertonic saline infusion to examine the effect of ibuprofen on renal handling of water and sodium during fasting. We measured the effect of ibuprofen/placebo on u-AQP2, u-ENaC_β_, fractional urinary excretion of sodium (FE_Na_), urinary excretion of prostaglandin E_2 _(u-PGE_2_), urinary excretion of cyclic AMP (u-c-AMP), free water clearance (C_H2O _), and plasma concentrations of renin (PRC), angiotensin II (p-Ang II), aldosterone (p-Aldo), vasopressin (p-AVP), atrial natriuretic peptide (p-ANP), and brain natriuretic peptide (p-BNP).

## Methods

### Participants

*Inclusion criteria*: Both males and females; age 18-65 years; body mass index <30. *Exclusion criteria: *Clinical signs or history of disease in the heart, lungs, kidneys or endocrine organs; abnormal laboratory tests (blood hemoglobin, white cell count, platelet counts, plasma concentrations of sodium, potassium, creatinine, albumine, bilirubine, alanine-aminotransferase, and cholesterol; blood glucose and albumin and glucose in urine); malignancies; arterial hypertension (i.e. casual blood pressure >140/90 mmHg); alcohol abuse (more than 21 drinks per week for males and more than 14 for females); medical treatment; pregnancy; breast-feeding; lack of oral contraceptive treatment to women in the fertile age; intercurrent diseases; problems with blood sampling or urine collection; allergy to ibuprofen; medicine abuse; donation of blood less than 1 month before the study; and unwillingness to participate. *Withdrawal criteria: *Development of one or more of the exclusion criteria.

### Ethics

The Scientific Committee of Ringkøbing, Ribe and Southern Jutland Counties (j.no.2623-04) approved the study. All participants received written information and gave their consent by signature. The study was carried out in compliance with the Helsinki Declaration.

### Design

The study was randomized, placebo-controlled, double blind, and over-crossed. There was a time interval of 2 weeks between the two examinations. Each examination took three days.

### Recruitment

Participants were recruited by advertisements in public and private institutions.

### Diet and fluid intake

The normal energy requirement was calculated with the formula: weight (kg) * 100 (kJ) * activity factor (AF). AF ranged from 1.3 to 2.4 with a possible extra of 0.3 for physical activity in the spare time, e.g. 30 min. of sport 5-6 times a week. AF of 1.3 indicates no physical activity. AF of 1.4-1.5 indicates sedentary work without physical activity in the spare time. AF of 1.6-1.7 indicates work with walking during work hour and/or physical activity in the spare time, AF of 1.8-1.9 corresponds to shop assistant, (standing/walk all day), AF 2.0-2.4 indicates hard physical activity with or without physical activity in the spare time. The food had a specified energy amount with carbonhydrates (55% of the total energy), protein (15% of the total energy), and fat (30% of the total energy). The diet contained three main meals and three small meals. The participants were not allowed to add any spices or sodium to the meals or to divide the meals into bigger or smaller portions. The participants drank tap water 35 mL/kg each 24-hour and nothing else. They maintained normal physical activity during the study.

### Procedure

Subjects were studied on three consecutive days. Table 1 shows the experimental design.

**Table 1:** Overview of the study design

	Day 1			Day 2			Examination day										
Time	7:00 AM	3:00 PM	11:00 PM	7:00 AM	3:00 PM	11:00 PM	7:00 AM	8:00 AM	9:00 AM	9:30 AM	10:00 AM	10:30 AM	11:00 AM	11:30 AM	12:00 AM	12:30 AM	13:00 AM

Food	specified diet			Fasting			Fasting										

Water	35 ml/kg body weight			35 ml/kg body weight			175 ml every ½ hour										

Ibuprofen/placebo				**x**	**x**	**x**	**x**										

Urine samples				24 hour urine collection			2 ½ hour urine collection				Baseline urine collection			1	2	3	4

Blood samples								↑	↑	↑	↑	↑	↑	↑	↑	↑	↑

3% saline infusion													7 ml/kg body weight				

*Day 1 *(24 hours): The participants ate the specified diet, drank tap water 35 mL/kg body weight, and maintained normal physical activity.

*Day 2 *(24 hours): The participants fasted, drank tap water 35 mL/kg body weight maintained normal physical activity, and collected urine during 24 hours. They received a tablet of 600 mg ibuprofen or placebo at 07.00 a.m., 03.00 p.m., 11.00 p.m., and the next morning at 07.00 a.m.

*Day 3*: The participants arrived at 7:30 a.m. in the laboratory. An intravenous catheter was placed in fossa cubiti on each side; one for collection blood samples, the other for infusion of the ^51 ^Cr-EDTA and hypertonic saline. Urine was collected from 7:30-9:30 to measure the effect variables after a period of 24 hours of fasting. Afterwards, urine was collected in the following seven periods: 09:30-10:00 a.m. (P-1), 10:00-10:30 a.m. (P-2), 10:30-11:00 a.m. (P-3), 11:00-11:30 a.m. (P-4), 11:30-12:00 a.m. (P-5), 12:00-12:30 p.m. (P-6) and 12:30-01:00 p.m. (P-7). Urine was analyzed for u-AQP-2, u-ENaC, u-Osm, u-Na, u-K, u-creat, u-cAMP, u-PGE_2_, u-^51^Cr-EDTA. The subjects voided in the standing or sitting position. Otherwise, they were in the supine position during the examination. Blood samples were taken every 30 min. starting at 09.30 a.m. for analysis of p-AVP, p-Osm, p-K, p-Na, p-Crea, p-albumin, p-^51^Cr-EDTA. In addition, blood samples for measurements of p-Ang II, p-ANP, p-BNP, p-PRC, and p-ALDO were drawn at 08.00 a.m., 11.00 a.m., 12.00 p.m., and 01.00 p.m. A total amount of 350 mL blood was drawn during the each study day. The blood drawn at blood sampling was immediately substituted with isotonic saline. From 11.00 a.m. to 11.30 a.m. (P-4), 3% saline was infused, 7 ml/kg body weight. Blood pressure and pulse rate were measured every 30 min. during the examination.

The participants were weighed before (day 3 at 07:30 a.m.) and after the trial (01:30 p.m.).

### Effect variables

Main effect variables were u-AQP2 and u-ENaC. The other effect variables were u-PGE_2_, u-C_AMP_, p-AVP, C_H2O _urine volume, FE_Na_, p-Osm, u-Osm, p-PRC, p-Aldo, p-ANP, p-BNP and p-Ang II.

### Number of participants

An increase in u-AQP2 of 0.2 ng/min was considered the relevant difference between ibuprofen and placebo. The standard deviation was estimated to be 0.15 ng/min. With a level of significance of 5% and a power of 90%, 14-15 healthy subjects needed to be included in the trial.

### Test substance

The Hospital Pharmacy conducted the randomization and prepared the medication. The tablets were kept in a sealed container with tablets for one day. Each tablet contained either ibuprofen 600 mg or placebo.

### Measurements

*Glomerular filtration rate *was measured using the constant infusion clearance technique with ^51^Cr-EDTA as a reference substance.

*U-AQP-2 *was measured by radioimmunoassay as previously described, and antibodies were raised in rabbits to a synthetic peptide corresponding to the 15 COOH-terminal amino acids in human AQP2 to which was added an NH_2_-terminal cystein for conjugation and affinity purification [10]. Minimal detection level was 32 pg/tube. The coefficients of variation were 11.7% (inter-assay) and 5.9% (intra-assay).

*U-c-AMP *was measured using a kit obtained from R & D Systems, Minneapolis, MN, USA. Minimal detection level was 12.5 pmol/tube. The coefficients of variation were 6.9% (inter-assay) and 5.3% (intra-assay).

*ENaC*_*β *_was measured by a newly developed RIA. Urine samples were kept frozen at -20°C until assayed. ENaC_β _was synthesized and purchased by Lofstrand Labs Limited - Gaithersburg, Maryland, USA, see Appendix. *U-PGE*_*2 *_was measured by a kit from Assay Designs, Inc., Ann Arbor, MI, USA. The coefficients of variations were 10.9% (inter-assay) and 6.3% (intra-assay).

Blood samples were centrifuged for 15 minutes at 3000 rpm at 4°C. Plasma was separated from blood cells and kept frozen at -20°C until assayed. *AVP, ANP, BNP, and Ang II *were extracted from plasma with C_18 _Sep-Pak (Water associates, Milford, MA, USA), and subsequently determined by radioimmunoassays [11,12]. The antibody against AVP was a gift from Professor Jacques Dürr, Miami, FL., USA. Minimal detection level was 0.5 pmol/L. The coefficients of variation were 13% (inter-assay) and 9% (intra-assay). Rabbit anti-ANP antibody was obtained from Department of Clinical Chemistry, Bispebjerg Hospital, Denmark. Minimal detection level was 0.5 pmol/L, coefficients of variation were 12% (inter-assay) and 10% (intra-assay). Rabbit anti-BNP antibody without cross-reactivity with urodilatin and α-ANP was used. Minimal detection level was 0.5 pmol/L plasma. The coefficients of variation were 11% (inter-assay) and 6% (intra-assay). The antibody against Ang II was obtained from Department of Clinical Physiology, Glostrup Hospital, Denmark. Minimal detection level was 2 pmol/L. The coefficients of variation were 12% (inter-assay) and 8% (intra-assay).

*Aldosterone i*n plasma was determined by radioimmunoassay using a kit from Diagnostic Systems Laboratories Inc., Webster, Texas, USA. Minimal detection level was 22 pmol/L. The coefficients of variations were 8.2% (inter-assay) and 3.9% (intra-assay).

*PRC *is determined by radioimmunoassay using a kit from CIS Bio International, Gif-Sur-Yvette Cedex, France. Minimal detection level was 1 pg/ml. The coefficients of variations were 14.5% (inter-assay) and 4.5% (intra-assay).

Plasma and urinary *osmolality *were measured by freezing-point depression (Advanced Model 3900 multisampling osmometer).

*Blood pressure *was measured with UA-743 digital blood pressure meter (A&D Company, Tokyo, Japan)

Plasma and urinary concentrations of *sodium and potassium *were measured by routine methods at the Department of Clinical Biochemistry, Holstebro Hospital, Denmark.

All clearances were standardized to a body surface area of 1.73 m^2^

### Statistics

Statistical level of significance was P < 0.05 in all analyses. We used A General Linear Model with Repeated Measures for comparison between ibuprofen treatment and placebo during fasting when several measurements were done during the examination. A paired t-test was used for comparison between two groups. Bonferroni correction was used when appropriate. Values are given as mean ± SD

## Results

### Demographics

Twenty subjects were allocated to the study. Three participants were excluded. The first was not able to fast for 24 hours, the second needed acute medication due to an intercurrent disease, and in the third blood sampling could not be performed. Seventeen participants were included in the study, 7 women and 10 men with a mean age of 33 ± 8 years. Blood pressure was 124/72 ± 14/9 mmHg. Blood samples showed: B-Hemoglobin 8.8 ± 0.79 mmol/L, P-Sodium 139 ± 2.4 mmol/L, P-Potassium 3.8 ± 0.3 mmol/l, P-Albumin 42 ± 1.5 g/L, P-Creatininium 78 ± 15 μmol/L, P-Bilirubin 8 ± 3 μmol/L, P-Alanintransaminase 23 ± 9 U/L, P-Glucose 5.2 ± 0.8 mmol/L, P-Cholesterol 4.4 ± 0.7 mmol/L.

### Effect of ibuprofen during fasting on u-AQP2, u-ENaC_β_, u-PGE_2_, u-c-AMP, C_H2O_, and p-AVP

Table 2 shows the effect of fasting during a 24 hours period and during 2½ hours immediately after 24 hours fasting. During fasting ibuprofen reduced u-PGE_2 _by 50% (P < 0.03), u-AQP2 by 17% (P < 0.01), and u-AQP2/u-Crea by 18% (P < 0.01). U-ENaC_β _was increased by 170% (P < 0.0001). We found no statistically significant changes in urine volume, C_H_2_O _and u-c-AMP despite a tendency to lower values during ibuprofen treatment.

**Table 2:** Effect of ibuprofen on urine volume, urinary excretion of prostaglandin E2 (U-PGE_2_), aquaporin 2 (U-AQP2), urinary epithelial sodium channel (U-ENaC_β _), cyclic AMP (U-C-AMP), free water clearance (C _H2O_), serum osmolality (s-Osm) and plasma arginine vasopressin (P-AVP) during 24 hours fasting and 2½ hours period immediately after 24 hours fasting in a randomized placebo-controlled, cross over study in 17 healthy subjects

	24 hours fasting urine collection			2½ hours urine collection immediately after 24 hours fasting		
	Ibuprofen	Placebo	P	Ibuprofen	Placebo	P
Urine volume (ml)	2533 (718)	2784 (770)	NS	2.75 (1.39)	3.30 (1.56)	NS
U-PGE_2 _(pg/min)	270.91 (95.4)	540.97 (365.1)	0.027	282 (111)	504 (307)	0.02
U-AQP-2 (ng/min)	1.21 (0.30)	1.48 (0.27)	0.00016	3.42 (2.07)	4.92 (3.09)	NS
U-AQP-2/U-Crea (ng/μmol)	128.9 (21.5)	158.0 (30.2)	0.00002	0.29 (0.17)	0.40 (0.18)	0.024
U-ENaC_β_/U-Crea (pg/μmol)	38.7 (13)	14.3 (3)	0.0000002	40.1 (11)	12.9 (4)	0.000000002
U-c-AMP (pmol/min)	5737 (2080)	6381 (3041)	NS	10909 (7061)	13762 (9571)	NS
C_H2O _(ml/min)	0.029 (0.47)	-0.047 (0.36)	NS	1.30 (1.40)	1.85 (1.29)	NS
Total Na^+ ^excretion (mmol/24 hour)	95 (44)	152 (33)	0.00001	x	x	x
FE_Na_	0.46(0.16)	0.71 (0.20)	0.000006			
S-Osm (mosm/kg H_2_O)	x	x	x	283 (2.6)	284 (4.1)	NS
P-AVP (pmol/l)	x	x	x	0.86 (0.21)	0.78 (0.20)	NS

Values are means and (SD). A paired t-test was used for comparison of means.

After fasting ibuprofen reduced u-PGE_2 _by 44% (P < 0.02), u-AQP2/u-Crea by 27% (P < 0.03), and u-ENaC_β _was increased by 210% (P < 0.0001) in 2½ hours urine collection immediately after the 24 hours fasting period. We measured no statistically significant changes in urine output, C_H_2_O _and u-c-AMP despite a tendency to lower values during ibuprofen treatment.

### Effect of ibuprofen on the response to hypertonic saline infusion

**Figure 1:**
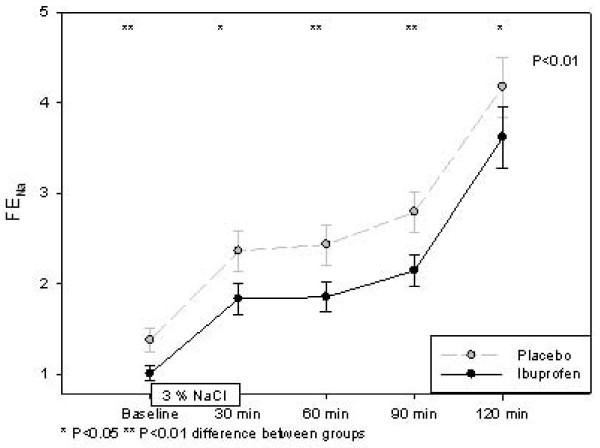
**Effect hypertonic saline infusion on ENaCb/crea in a randomized placebo-controlled, cross-over study of 17 healthy subjects**.

Table 3 shows the effect variables before, during and after hypertonic saline infusion. U-AQP2, p-AVP, u-Osm and p-Osm increased significantly during infusion of 3% saline. After 120 min. U-AQP2 increased by 29% (p < 0.01) during ibuprofen treatment and by 51% (p < 0.001) during placebo. The level of u-AQP2 was significantly lower during ibuprofen treatment at three of four measurements after 3% saline infusion. During ibuprofen treatment we found that ENaC_β _was increased significantly and approximately 3 fold at all times during the hypertonic saline infusion (figure 1). After 60 min we found a modest, but significant reduction in ENaC_β _by 12% (p < 0.001), and this reduction remained unchanged during the study. During placebo ENaC_β _levels were reduced significantly at 30 min and again 120 after hypertonic saline infusion. P-AVP increased by 108% (p < 0.001) immediately after 3% saline infusion in the ibuprofen group and by 118% (P < 0.001) in the placebo group. U-Osm increased by164% (p < 0.001) immediately after 3% saline infusion in the ibuprofen group and by 157% (P < 0.001) in the placebo group. S-Osm increased 3% (p < 0.001) immediately after 3% saline infusion in the ibuprofen group and by 2.5% (P < 0.001) in the placebo group. The levels of P-AVP, u-Osm and s-Osm did not differ between ibuprofen and placebo. Urine volume and C_H2O _decreased by 55% (p < 0.001) and 105% (p < 0.001), respectively, during ibuprofen administration and by 57% (p < 0.001) and 109% (p < 0.001) during placebo. Urine volume and C_H2O _did not differ significantly between the two groups. U-PGE_2 _did not change significantly during 3% saline infusion. The initial reduction in u-PGE_2 _persisted during ibuprofen treatment both during and after 3% saline infusion. GFR and u-c-AMP did not change significantly during 3% saline infusion and did not differ between ibuprofen and placebo treatment.

**Table 3:** Urinary aquaporin-2 creatinine adjusted (U-AQP2/u-crea), urinary epithelial Na^+ ^channel (U-ENaCβ/u-crea), plasma arginine vasopressin (AVP), urine osmolality (U-OSM), serum osmolality (S-OSM), urinary excretion of prostaglandin2 (U-PGE2), free water clearance (CH2O), urinary excretion of cAMP (U-CAMP), and Glomerular filtration rate (GFR).

		Baseline	30 min	60 min	90 min	120	P
U-AQP2/u-crea (ng/μmol)							
	Placebo	154(33)	189(69) **	200(61) ***	225(75) ***	233(76) ***	0.005
	Ibuprofen	161 (25)	161(62)	202(68)*	199(52)**	208(64)**	
	P-value	NS	0.036	NS	0.008	0.014	

U-EnaC/u-crea (pg/μmol) Placebo		11.6(3)	13.4(3) **	12.8(4)	13.4(4)	13.7(4) **	< 0.0001
	Ibuprofen	44.8(13)	44.1 (10)	38.6 (10) **	39.1 (10) **	35.8 (9) ***	
	P-Value	<0.0001	<0.0001	<0.0001	<0.0001	<0.0001	

P-AVP (pg/ml)							
	Placebo	0.87(0.5)	1.9(0.13) ***	1.5(0.12) ***	1.3(0.13) ***	1.2(0.9) ***	NS
	Ibuprofen	0.91(0.5)	1.9(0.15) ***	1.5(0.11) ***	1.4(0.10) ***	1.2(0.9) ***	

U-OSM (mosm/kg H2O)							
	Placebo	130(25)	335(77) ***	588(113) ***	585(11) ***	566(110) ***	NS
	Ibuprofen	125(31)	331(107) ***	631(201) ***	625(119) ***	612(130) ***	

S-OSM (mosm/kg H2O)							
	Placebo	283(2.7)	291(4.1) ***	290(3.2) ***	289(3.6) ***	287(3.7) ***	NS
	Ibuprofen	283(3.0)	293(3.7) ***	291(2.6) ***	289(3.2) ***	287(3.5) ***	

U-PGE2 (pg/min)							
	Placebo	281(123)	212(105)	256(118)	293(133)	298(173)	<0.001
	Ibuprofen	507(341)	385(180)	415(138)	406(160)	507(411)	
	P-Value	0.02	0.001	0.0004	0.005	0.02	

CH2O (ml/min)							
	Placebo	4.05(1.2)	-0.38(0.5) ***	-1.60(0.6) ***	-2.00(0.7) ***	-2.00(0.7) ***	NS
	Ibuprofen	3.70(1.4)	-0.20(1.0) ***	-1.62(0.8) ***	-1.92(0.7) ***	-2.00(0.7) ***	

Urine (ml/min)	Placebo	7.02(1.57)	3.0(0.19)***	1.8(0.20)***	2.16(0.20)***	2.3(0.20)***	<0.0001
	Ibuprofen	6.31(1.71)	2.8 (0.23)***	1.6(0.12)***	1.7(0.11)***	1.9(0.13)***	
	P-Value	NS	NS	NS	0.016	NS	

U-CAMP (pmol/min)							
	Placebo	3819(1034)	3974(1251)	4285(1387)	4114(1353)	4219(1125)	NS
	Ibuprofen	4016(1180)	3738(759)	4447(1243)	4196(1248)	4377(1136)	

GFR (ml/min)							
	Placebo	98(12)	94(16)	94(21)	103(21)	102(17)	NS
	Ibuprofen	98(13)	93(18)	98(15)	101(16)	103(12)	

Baseline values before hypertonic saline infusion, 30, 60, 90 and 120 min after hypertonic saline infusion in 17 healthy humans administered with ibuprofen or placebo.

Values are means and (SD). P indicates significant difference between ibuprofen treatment and placebo using a General Linear Model With Repeated Measures. A paired t-test was used for comparison of means, when differences were found between the two treatments. *indicated a statistical significant changed from baseline values* P < 0.05 ** P < 0.01, *** P < 0.001.(longitudinal comparison)

### Effect of ibuprofen on FE_Na _and total sodium excretion

**Figure 2:**
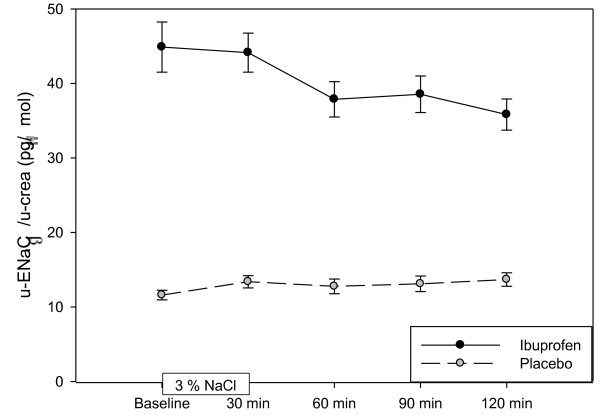
**Effect hypertonic saline infusion on fractional sodium excretion (FENa) in a randomized placebo-controlled, cross-over study of 17 healthy subjects**.

During 24 hours of fasting the total sodium excretion was reduced by 37% (p < 0.001) during Ibuprofen treatment, and FE_Na _was reduced by 35% (p < 0.001). figure 2 shows that ibuprofen reduced FE_Na _significantly at baseline, during and after hypertonic saline infusion. The changes after 3% saline infusion were the same in the two groups, but at different levels. FE_Na _at baseline was 36% (p = 0.006) higher during placebo treatment compared with ibuprofen, and this difference remaind after infusion of hypertonic saline. Urinary sodium excretion increased significantly from baseline (Placebo: 1129 ± 509 μmol/min; Ibuprofen 827 ± 541 μmol/min) to the level during infusion (Placebo: 2856 ± 1068 μmol/min; Ibuprofen 3305 ± 941 μmol/min) and in the period after infusion (Placebo: 5603 ± 1907 μmol/min; Ibuprofen 5978 ± 1793 μmol/min).

### Effect of ibuprofen on vasoactive hormones after 24 hours fasting and after hypertonic saline infusion

Ibuprofen treatment increased p-BNP significantly during all periods of the study, except for 60 min after infusion (Table 4). P-BNP increased by 61% (p < 0.03) immediately after 24 hours of fasting by ibuprofen. At baseline (before saline infusion) p-BNP was increased by 60% (p < 0.01). No significant increase in p-BNP values there observed 6o min after hypertonic saline infusion, but a clearer tendency was seen to a higher levels during ibuprofen (p < 0.08). The increase in p-BNP was 42% 120 min after hypertonic saline infusion (p < 0.02). P-ANP tended to increase using A General Linear Model With Repeated Measurements (p < 0.055). At baseline, P-ANP increased by 29% (p < 0.03), and 60 min after hypertonic saline infusion the increase was 20% (p < 0.03). No significant difference was observed 120 min after hypertonic saline infusion. Both p-Ang II and p-aldo tended to fall during ibuprofen treatment.

**Table 4:** Effect of Ibuprofen on plasma atrial natriuretic peptide (p-ANP), plasma brain natriuretic peptide (p-BNP), plasma angiotensin II (p-AngII) and p-aldosterone (p-Aldo) at morning after 24 hours of fasting (morning), before hypertonic saline infusion (baseline), 60 min. after hypertonic saline infusion (60 min. post) and 120 min. after hypertonic saline infusion (120 min. post) compared with placebo in a randomized clinical trial with17 healthy subjects

		Morning	Baseline	**60 min. post**.	**120 min. post**.	P
p-ANP (pmol/l)						
	Placebo	5.95(3.37)	5.75(2.68)	9.84 (3.89)	8.39(4.03)	NS
	Ibuprofen	6.80(3.22)	7.44(3.46)	11.85(5.54)	9.13 (3.78)	

p-BNP (pmol/l)						
	Placebo	1.59(1.10)	1.65(0.99)	2.66 (2.26)	2.17 (1.55)	0.015
	Ibuprofen	2.57(2.08)	2.65(2.03)	3.44 (3.27)	3.09 (2.38)	
	p-value	0.025	0.004	NS	0.015	

p-Renin (mU/l)						
	Placebo	10.1(5.3)	12.2(7.8)	9.5(14.7)	5.5 (2.1)	NS
	Ibuprofen	10.7(12.3)	13.6(22.1)	12.5 (21.3)	4.9 (2.5)	

p-AngII(pmol/l						
	Placebo	8.2 (3.0)	10.5 (5.9)	6.5(4.4)	6.2 (3.5)	NS
	Ibuprofen	7.1 (3.4)	7.6 (4.6)	5.9 (4.0)	5.2 (2.7)	

p-Aldo (pmol/l)						
	Placebo	310.8 (211.6)	230.4 (145.5)	164.7 (63.0)	162.4 (57.1)	NS
	Ibuprofen	293.9 (137.6)	190.9 (55.6)	156.6 (27.3)	161.6 (36.6)	

Values are means and (SD). P indicates significant difference between ibuprofen treatment and placebo using a General Linear Model With Repeated Measures. A paired t-test was used for comparison of means, when differences were found between the two treatments.

### Effect of ibuprofen on blood pressure

At baseline blood pressure was 115/64 ± 9/8 mmHg and pulse rate was 57 ± 8 beats/min during Ibuprofen treatment. The corresponding values during placebo did not deviate significantly, blood pressure was114/65 ± 11/7 mmHg and pulse rate 56 ± 7 beats/min.

The response to 3% saline infusion was measured 30 min after infusion. Systolic blood pressure was unchanged during ibuprofen treatment and increased by 4 mmHg (p < 0.01) during placebo. Pulse rate increased by 11 beats/min (p < 0.01) during ibuprofen treatment and 9 beats/min (p < 0.01) during placebo. Diastolic blood pressure decreased by 2 mmHg (p < 0.05) during ibuprofen treatment and were unchanged during placebo.

The response to 3% saline infusion was measured 60 min after infusion. Systolic blood pressure was unchanged during both ibuprofen and placebo treatment. Pulse rate was significantly increased in both groups by 7 Beats/min (p < 0.01) during ibuprofen and 6 beat/min (p < 0.01) during placebo. Diastolic blood pressure was decreased in both groups (p < 0.05). There were no statistical differences in blood pressure and pulse rate between ibuprofen and placebo at baseline, during and after 3% saline infusion.

### Effect of ibuprofen and fasting on body weight and p-albumin

Body weight were 77.6 kg in the placebo group and 78.1 kg in the ibuprofen group (P < 0.07) after the study day, at the same time there was a decrease in p-albumin from 41 g/l at baseline to 37 g/l (p < 0.001) at the end of the study. None of the subjects had edema after infusion of 3% sodium chloride.

## Discussion

The present study of healthy subjects showed that ibuprofen decreased FE_Na _and increased u-ENaC_β _both during a 24 hours fasting period, during the first two hours after 24 hours fasting, and during the following period with hypertonic saline infusion. The increased tubular sodium absorption during ibuprofen treatment might be attributed to increased sodium transport from the tubular lumen via the epithelial sodium channels. Our hypothesis was not falsified regarding this point. However, ibuprofen decreased u-AQP2 and u-PGE_2_, and did not change p-AVP, u-c-AMP, urinary output, and free water clearance during any of the study periods. Thus, our results demonstrated that u-AQP2 was reduced, although an increased AQP2 trafficking might be expected, when the antagonizing effect of prostaglandins on the vasopressin mediated effect on the principal cells was reduced/eliminated during ibuprofen treatment. Consequently, our hypothesis that reduction of prostaglandin synthesis would increase u-AQP2 was falsified.

In the present study we have used a new and original method to evaluate sodium reabsorption in the principal cells in the distal tubules using a radioimmunoassay of the β-fraction of the protein in the epithelial sodium channel. The amount of ENaC_β _in urine is supposed to reflect the activity of sodium transport via the epithelial sodium channels just as u-AQP2 reflects the functional status of the AQP2 water channels (31). Our analyses showed that the assay has a satisfactory reliability. In addition, we demonstrated a significant correlation between changes in urinary sodium excretion and changes in u-ENaC_β_. Thus, our results are in accordance with u-ENaC_β _being a biomarker of the transport of sodium via ENaC during acute studies, presumably reflecting up- and down regulation β-ENaC expression and sodium transport via ENaC. It is well known that ibuprofen reduces renal sodium excretion. Our study adds new information, since our data might suggest that prostaglandin inhibition possibly reduce renal sodium excretion via an increased transport through ENaC.

In our study, ibuprofen significantly reduced FE_Na _during all parts of study to a level around 35% lower than placebo. The response in FE_Na _during hypertonic saline infusion was the same during treatment with ibuprofen and placebo, and the difference, i.e. the lower level of FE_Na _during ibuprofen treatment, persisted during the infusion. Several animal studies have shown that PGE_2 _has a direct inhibitory effect on sodium chloride transport in the collecting ducts [2,5,13,14]. In rats, this is regulated by the EP2 receptor[15]. We found that u-ENaC_β _was significantly increased during ibuprofen treatment. Thus, the decrease in FE_Na _might, at least partly, be explained by an increased transport of sodium from the tubular lumen via ENaC. However, we cannot exclude that the antinatriuretic effect of ibuprofen was also attributed to some extent by an upregulation of the Na-K-2Cl cotransporter in the ascending limb of Henle's loop [16]. U-ENaC_β _was increased by ibuprofen without an increase in either p-AVP or u-c-AMP, which indicates that AVP does not seem to play a direct regulatory role in the increased u-ENaC_β_. However, the marked reduction in u-PGE_2 _makes it likely that the reduced prostaglandin level results in the increase sodium transport via ENaC, and this explanation is supported by evidence from animal studies [7,17-19].

Ibuprofen did not change the response in the effect variables qualitatively during hypertonic saline infusion, but renal sodium excretion was reduced as hypothesized. After hypertonic saline infusion u-ENaC was reduced significantly at 60 minutes, and at the same time p-ANP tended to increase. An increase in ANP might be responsible for reduction in u-ENaC_β _during this condition, and could be seen as a homeostatic mechanism to prevent sodium and fluid expansion [6]. Aldosterone is an important regulator of the transport activity via ENaC. Aldosterone stimulates the MR receptor. The result is an increased transcription of genes, which code for proteins involved in sodium transport, i. e. ENaC and Na-K-ATPase [3,20]. In-vitro and in-vivo studies have shown that aldosterone increases the synthesis of the α-fraction of ENaC in the distal tubuli [21].

In our study, we found that u-ENaCβ was reduced in the ibuprofen group during and after hypertonic saline infusion. We did not measure a reduction in p-aldosterone. This might be due to the fact that our study was too short to allow the regulatory effect of aldosterone.

A time delay in aldosterone secretion has previously been demonstrated in another study, in which an acute infusion of hypertonic saline infusion decreased PRA, but plasma aldosterone was unchanged [22]. We think the short observation time after the hypertonic saline infusion might explain the fact that p-aldosterone was unchanged in the present study.

During placebo treatment, hypertonic saline resulted in a significant increase in u-ENaCβ. This increase was associated with an increased FE_Na_. The explanation of this phenomenon is not clear for the time being. A considerably decrease in the renal sodium absorption more proximally in the nephron might be compensated for by an increase in absorption in the distal part of the nephron, but additional studies are required to determine the precise use of u-ENaCβ as biomarker for ENaC activity during these conditions. However, a rise in the intracellular sodium concentration was suggested to be a trigger for the feedback inhibition of sodium absorption via ENaC in rats when expressed in xenopus laevis oocytes [23]. Thus, the reduction in u-ENaC_β _during sodium loading could alternatively be attributed to this inhibitory feedback mechanism of increased intracellular sodium on sodium transport via ENaC.

In a previous study, we showed that fasting decreased u-AQP2 and reduced the stimulating effect of vasopressin on u-AQP2 [24]. Fasting increased prostaglandin synthesis, and the refractoriness to vasopressin during fasting was proposed to be due to an antagonizing effect of prostaglandins on AQP2 trafficking. Thus, it would be reasonable to hypothesize that an increase in u-AQP2 could be expected by ibuprofen treatment. On the contrary, ibuprofen reduced u-PGE_2 _markedly in the present study during all conditions i.e. in 24 hours urine, in the period shortly after 24 hours of fasting, and during infusion of hypertonic saline, as expected. However, ibuprofen also reduced u-AQP2 during all parts of the study, and thereby falsified our hypothesis that reduction of prostaglandin synthesis would increase u-AQP2.

A previous study in humans showed that prostacyclin infusion increased u-AQP2 excretion, and this seems to be in agreement with our results [25]. Apparently, a discrepancy exists between animal studies on the one hand and studies in man on the other. In animals, prostaglandin decreased APQ2 trafficking [2,26], and inhibition of prostaglandin synthesis by infusion of indomethacin increased urinary excretion of AQP2 [4]. In healthy man prostaglandin and prostaglandin-inhibitors had the opposite effects, as demonstrated in the present study and by others [25]

We found that ibuprofen significantly reduced u-PGE_2 _due to a reduced renal prostaglandin synthesis. This is in accordance with other studies in healthy man [27,28]. Prostaglandin E_2 _increased urinary output and sodium excretion by inhibition of AVP stimulated water absorption, inhibition of sodium absorption and stimulation of basal water absorption [8]. Different subtypes of prostaglandin receptors mediate this effect from the basolateral part of the tubular cells [29]. Receptor stimulation by PGE_2 _reduced sodium reabsorption in the thick ascending limb of Henle and in the cortical collecting duct, and reduced AVP-induced water absorption in cortical collecting duct [9,30,31]. Both p-AVP and u-c-AMP were unchanged by ibuprofen, but simultaneously we measured a clear reduction in u-AQP2. Thus, the vasopressin-c-AMP axis was not involved in the reduction in u-AQP2. It is generally accepted that prostaglandin E_2 _antagonizes the effect of vasopressin on AQP2 trafficking via the EP_3 _receptor. Activation of this receptor inhibits adenylyl-cyclase, reduces the level of c-AMP, causing increased urinary output. However, the close relationship between c-AMP production and increased AQP2 trafficking was challenged in a recent experimental study [32]. Activation of the EP3 receptor inhibited AQP2 trafficking in inner medullary cells from rats, despite high levels of c-AMP, probably due to an cAMP- and Ca2+-independent Rho activation. Rho promotes the formation F-actin which hinders AQP2 coated vesicles reaching the apical membrane [32]. Our study in healthy man is in agreement with these findings, in the sense that we found a reduction in u-AQP2 excretion without changes in urinary excretion of c-AMP. This suggests that adenylyl cyclase activity did not contribute to our results regarding u-AQP2 excretion. The changes in p-AVP during hypertonic saline infusion was as expected, and the changes were not significantly different during ibuprofen treatment compared with placebo.

As expected, we measured a marked reduction in urinary sodium excretion during ibuprofen treatment as reflected in the decrease in FE_Na_, but no significant changes in urinary output and free water clearance. However, some tubular reabsorption of water must take place simultaneously with sodium reabsorption due to the effect of ibuprofen on basal renal water transport. Most likely, this fact explains our findings, i.e. the lack of increase in urinary output and free water clearance despite decreased u-AQP2 during ibuprofen treatment. Both p-AVP and u-c-AMP were unchanged by ibuprofen, but simultaneously we measured a clear reduction in u-AQP2. Thus, the vasopressin-c-AMP axis was not involved in the reduction in u-AQP2. The changes in p-AVP during hypertonic saline infusion was as expected, and the changes were not significantly different during ibuprofen treatment compared with placebo.

However, other regulatory factors than vasopressin and prostaglandins may influences the expression of AQP2 and u-AQP2 such as the renin-angiotensin-aldosterone system, the natriuretic peptide system, and the sympathetic nervous system [1,6,33-38]. We measured higher levels of p-BNP and a clear tendency to an increase in p-ANP simultaneous with a tendency to a lower level of p-Ang II and p-Aldo during ibuprofen treatment. Most likely, these changes in hormones with both vasoactive- and sodium- and water regulating properties are secondary to the sodium retention induced by ibuprofen. This is supported by the fact that p-albumin fell significantly, and that body weight tended to increase, presumably due to an expansion of the extracellular fluid volume. Animal studies support this explanation. Angiotensin II stimulated/enhanced AQP2 expression [36,38], and angiotensin II receptor blockade reduced AQP2 expression [1]. Aldosterone agonism and antagonism increased and decreased AQP2 expression, respectively [34]. We suggest that the tendency to reduced levels of the components in the renin-angiotensin-aldosterone system contributes to the reduced level of u-AQP2 during ibuprofen treatment in fasting healthy humans.

The role of ANP in the regulation of intracellular distribution of AQP2 was addressed in rats [6]. ANP-infusion had no immediate effect on the intracellular localization of AQP2, but after 90 minutes of ANP-infusion an increased apical targeting of AQP2 was noted. This was regarded as either a direct or compensatory effect to volume depletion to avoid dehydration. A human study with head out water immersion demonstrated increased AQP2 expression accompanied by an increase in p-ANP [33]. These findings do not prove any causal relationship, as several other homeostatic systems as the sympathetic nervous system and the renin-angiotensin-aldosterone system are also influenced by the intervention. Thus, the effect of the natriuretic peptide system on AQP2 trafficking and urinary excretion is not fully elucidated. Accordingly, we cannot rule out that the increased levels of the natriuretic peptides after ibuprofen treatment had modulated u-AQP2 in our study.

Inhibition of the renal prostaglandin synthesis might be dangerous in patients with heart failure, lever disease and renal insufficiency. It can result in sodium and water retention and hypertension. We did not measure any changes in blood pressure and the increase in body weight was marginal. Most like, this can be attributed to the fact that we studied healthy subjects.

## Conclusions

In conclusion, ibuprofen decreased urinary sodium excretion considerably in healthy man during fasting, a state with an increased prostaglandin synthesis. During the reduced prostaglandin synthesis u-ENaC_β _was markedly increased. Our results suggest that prostaglandins have an important direct regulatory function on ENaC trafficking. During hypertonic saline infusion, angiotensin II and aldosterone tended to decrease in plasma and the natriuretic peptides increased, which presumably can be seen as compensatory phenomena to prevent extracellular fluid expansion. Surprisingly, ibuprofen also decreased urinary u-AQP2 both during 24 hours of fasting and during hypertonic saline infusion. This effect was not mediated via the vasopressin-c-AMP-axis, but may be mediated by the changes in the natriuretic peptide system and/or the angiotensin-aldosterone system.

## Competing interests

The authors declare that they have no competing interests.

## Authors' contributions

All authors have made substantial contribution in designing the study and collection of data. They have all contributed in writing the manuscript and have approved the final version.

## Appendix

Description of the u-ENaC_β _analysis, *ENaC*_*β *_was measured by a newly developed RIA. Urine samples were kept frozen at -20°C until assayed. ENaC_β _was synthesized and and purchased by Lofstrand Labs Limited - Gaithersburg, Maryland, USA.

The β-ENaC antibody was raised against a synthethic peptide in rabbits and affinity purified as previously described [39] Iodination of ENaC_β _was performed by the chloramine T method using 40 μg of ENaC_β _and 37 MBq ^125^I. The reaction was stopped by addition of 20% human serum albumin. ^125^I-labeled ENaC_β _was separated from the iodination mixture by the use of a Sephadex G-25 Fine column. The assay buffer was 40 mM sodium phosphate (pH = 7.4), 0.2% human albumin, 0.1% Triton X-100, and 0.4% EDTA. A 1.5% solution of gamma globulins from pig (Sigma) and 25% polyethylene glycol 6000 (Merck) also containing 0.625% Tween 20 (Merck) was prepared using the 0.4 M phosphate buffer. Urine samples were kept frozen at - 20°C. After thawing out urine samples were centrifuged for 5 min at 1.6 × 100 *g *(3,000 rpm). The supernatant was extracted using Sep-pak C_18_. The eluation fluid was 4 ml of a mixture comprising 90% methanol, 0.5% acetic acid and 9.5% demineralized water. The eluates were freeze-dried and kept at - 20°C until assayed. The mixture of 300 μl of standard or freeze-dried urine eluates redissolved in 300 μl assay buffer and 50 μl of antibody was incubated for 24 h at 4°C. Thereafter, 50 μl of the tracer were added, and the mixture was incubated for a further 24 h at 4°C. Gamma globulin from pigs (100 μl) and 2 ml polyethylene glycol 6000 were added. The mixture was centrifuged at 3,500 rpm for 20 min at 4°C. The supernatant (free fraction) was poured off, and the precipitate (bound fraction) was counted in a gamma counter. The unknown content in urine extracts was read from a standard curve. For 13 consecutive standard curves, the zero standard was 70 ± 1.6%, and for increasing amounts of ENaC_β _standard the binding inhibition was: 69 ± 1.4% (15.6 pg/tube), 66 ± 1.5% (31.25 pg/tube), 62 ± 1.6% (62.5 pg/tube), 54 ± 1.5% (125 pg/tube), 40 ± 1.4% (250 pg/tube), 26 ± 1.2% (500 pg/tube), 14 ± 0.6% (1000 pg/tube), 8.2 ± 0.4% (2000 pg/tube), and 5.1 ± 0.3% (4000 pg/tube). The ID 50, i.e. the concentration of standard needed for 50% binding inhibition was 322 ± 12 pg/tube (n = 13). The nonspecific binding determined by performing the RIA without antibody was 1.3 ± 0.3% (n = 13). The inter-assay variation was determined by quality controls from the same urine pool spiked with ENaC_β _standard. In consecutive assays the coefficient of variation was: at a mean level of 78 pg/tube 12% (12 assays), at a mean level of 155 pg/tube 10% (12 assays), and at a mean level of 394 pg/tube 17% (10 assays). The intra-assay variation was determined on samples from the same urine pool in several assays at different concentration levels. At a mean level of 180 pg/tube (*n *= 10) and 406 pg/tube (n = 10), the coefficients of variation were 6.4% and 9.0%, respectively. In addition, coefficients of variation were calculated on the basis of duplicate determinations in different assays to 9.1% (n = 22) in the range 58-101 pg/tube, 8.6% (n = 26) in the range 143-203 pg/tube, 8.7% (n = 20) in the range 205-421 pg/tube, and 10.0% (n = 68) in the whole range 58-421 pg/tube. The sensitivity calculated as the smallest detectable difference at the 95% confidence limit was 10 pg/tube in the range 58-101 pg/tube (n = 22), 20 pg/tube in the range 143-203 pg/tube (n = 26), 48 pg/tube in the range 205-421 pg/tube (n = 20), and 28 pg/tube in the whole range 58-421 pg/tube (n = 68). The lower detectable limit of the assay was 34 pg/tube. It was calculated using the average zero binding for 13 consecutive assays minus 2 SD. The volume of urine used for extraction from the same pool was varied (18 different volumes in the range 250-6000 μl), and the mean concentration measured was 89 ± 6 pg/ml. There was a highly significant correlation between the extracted volume of urine and the amount of pg/tube (r = 0.99, n = 18). Recovery of the labeled tracer during the extraction-freezing drying procedure was 94 ± 3% (n = 13), 95 ± 3% (n = 13), 95 ± 2% (n = 10), and 95 ± 2% (n = 7) in four different pools used in several extraction procedures. When ENaC_β _in the range 62.5 to 250 pg was added to urine, a highly significant correlation was found between the measured and the expected values (r = 0.981, n = 12, P < 0.001). We measured u-ENaC_β _in 9 patients with arterial hypertension treated with amiloride. During the study day, patients collected a urine sample at 08.00 and 11.00. They took no medication in the morning before the first urine sample. Immediately after the first urine sampling, the patients took their usually dosis of amiloride 5 or 10 mg. For the whole group a significant correlation was found between the changes in u-Na/Crea and changes in u-ENaC_β_/crea (ρ = -0.720, n = 9, P = 0.029)

## Pre-publication history

The pre-publication history for this paper can be accessed here:

http://www.biomedcentral.com/1471-2369/11/28/prepub
